# Seven common mistakes to avoid in achieving long‐term success with dermatology patients

**DOI:** 10.1002/vms3.1

**Published:** 2015-03-04

**Authors:** Lowell Ackerman

**Affiliations:** ^1^ Westborough Boston Massachusetts USA

**Keywords:** dermatology, atopic dermatitis, demodicosis, pyoderma, compliance, adherence, standard of care, consistency of care, care pathway, protocol, evidence‐based medicine

## Abstract

Managing a pet's chronic dermatologic conditions can be frustrating for both clients and clinicians, but there are steps to making the process easier for everyone. Avoid the following seven mistakes sometimes made in managing dermatology cases and you will find the process considerably easier in the future.

## Not appreciating appropriate clinical timelines

While most veterinarians focus on the needs of the client and pet at the time of a specific office visit, it is important to realize that for many dermatologic conditions opportunities exist on a time continuum for intervention and client education. In some instances, the first discussions with the owner may take place long before there is even a skin problem to treat. So, for the owner of a breed at risk for atopic dermatitis, discussions should actually start during puppyhood, long before there might be any clinical evidence of allergies (Ackerman 2011). This is the best time to instruct owners to be vigilant looking for early signs of disease, such as licking and chewing at the feet, ear infections, or rashes in the axillary and inguinal areas. Perhaps more importantly, this provides an opportunity to discuss that if atopic dermatitis is eventually diagnosed in the pet that this is a lifelong condition without a cure, but early detection provides the most options for successful long‐term management. This might also prompt a discussion of the likely costs associated with long‐term management, so the owners might consider their options for risk management strategies, including pet insurance.

Appreciating appropriate timelines also helps in treatment selection. When a two‐year‐old atopic patient presents with pruritus, the first tendency might be to treat with glucocorticoids (corticosteroids) to achieve short‐term relief. However, when you reflect on how that same patient might look at 8 or 10 years of age with such treatment, there is often a different perspective on what the long‐term liabilities are for such short‐term gains, and alternative more targeted treatments might be selected, given the need for sensible long‐term solutions.

It is also important to schedule effectively. No one can solve a complicated dermatology case in a single visit, regardless of the appointment's duration, or the clinician's expertise. You can address the client's primary concerns, but be clear with the client that the pet's problems will likely not be solved with that single visit. The key to successful dermatology management is scheduling frequent re‐evaluation appointments. The success of this strategy is predicated on sharing the action plan with pet owners, so they can understand and appreciate what is expected to be accomplished, and according to what timeline.

For a young dog with localized juvenile demodectic mange, the owner should expect regular re‐evaluations over a several‐month period. They also need to realize that the underlying problem could be some form of immune dysfunction, which might be transitory or long‐lasting. Regardless, the condition is characterized by the overpopulation of mites normally resident in the skin (these are not external invaders), which in turn typically causes bacterial infections in the hair follicles, and the sequel to that is inflammatory changes and hair loss. The initial goal might be to control the associated bacterial folliculitis, provide supportive care in terms of frequent shampooing with a follicular‐flushing agent, ensure there is no evidence of other internal or external parasites, assess immune function, and put the pet on an improved plane of nutrition. Actually killing the mites might not even be necessary as a first treatment attempt. If the owner realizes that a cure without miticidal therapy is possible because many young animals ‘self‐cure’ with appropriate supportive therapy, they are more likely to understand why you are approaching the problem in stages, rather than just reaching for the strongest mite‐killing therapy on the shelf. Miticidal therapy can always be used if this otherwise healthy young animal fails to self‐cure in a reasonable amount of time. The pathway to resolution might include supportive care, decreasing mite numbers, resolution of the secondary bacterial folliculitis and, finally hair regrowth. It is also important for owners to realize that clinical cure often precedes microscopic cure, so it is important to continue to monitor with recheck examinations and skin scrapings before stopping therapy prematurely; otherwise, recurrence is more likely. When owners understand the plan, they can also appreciate the timeline to achieve it.

There are many dynamics at work in the development and resolution of skin problems and they can either work for or against the clinician depending on how they are relayed to pet owners.

The skin is not a sterile surface and is home to a large population of resident organisms. There are also lots of occasions for the skin to be colonized by the opportunistic overgrowth of organisms typically present in small numbers, the most common of which are bacteria (such as *Staphylococcus pseudintermedius*) and yeasts (such as *Malassezia pachydermatis*). As it is neither possible nor desirable to try to sterilize the skin, the goal needs to be to address the underlying reason for the microbial overgrowth (allergies, hypothyroidism, immune dysfunction, skin folds, etc.), manage it effectively (possibly long term) and use sensible systemic and topical therapies to help curb the microbial populations. The population dynamics also dictates reasonable treatment intervals. If staphylococcal populations can double in number within minutes to hours on the skin surface, can an every‐other‐week bathing schedule ever have much of an impact? Probably not. If owners want to help control surface bacterial numbers with topical therapies, they need to realize that more frequent treatments are likely needed, at least initially.

Similarly, when it comes to epidermal turnover rates in keratinization disorders (e.g. seborrhea), we are at the mercy of those skin cell dynamics. Even if you could wave a magic wand and immediately correct the problem, it would still take weeks for the ‘normal’ skin to grow through the epidermal layers and replace the abnormal skin cells on the skin surface. Without a magic wand it takes many more weeks and even months for progress to be noted. When clients realize this, it helps them be more realistic with their expectations.

## Not appropriately framing client expectations

Many pet skin problems cannot be cured, regardless of the medication selected, so set realistic expectations with clients early in the process. Fortunately, control of the condition and a better quality of life for the pet can be attained with appropriate management. For the pet with atopic dermatitis, you might explain to clients that this is no different than addressing other incurable but manageable medical conditions such as diabetes mellitus or degenerative joint disease (osteoarthritis). We can often achieve excellent long‐term control with ongoing treatment and close monitoring, but it is unlikely that we will be able to ‘fix’ the underlying problem. Usually once owners appreciate that the goal is control rather than cure, their expectations tend to be more reasonable and realistic.

Similarly, it is important to manage expectations around the metrics of treatment success and the timeframe in which such judgment can be anticipated. For example, in a dog with demodicosis and associated hair loss, the pet owner may judge success as hair regrowth, while the clinician will likely be more focused on controlling mite numbers initially. So, how long after treatment initiation should the client expect to see hair regrowth if the treatment is working? If the owners are told to expect the likely onset of hair regrowth eight weeks or so after the mite population has been satisfactorily reduced, they will not be expecting that hair growth shortly after starting treatment; hopefully they will appreciate that mite population control must happen first, before hair regrowth can start. They should also then be attentive at recheck visits and use progress on mite control reported by the clinician as a signpost for the path to eventual resolution (and hair regrowth).

For the dog with sarcoptic mange, it is important to notify the owner that the pruritus is likely to persist for another week or two even after successful treatment has been initiated. This occurs because mite faecal pellets that remain in the epidermal burrows still trigger hypersensitivity reactions, and because dead and decaying mite fragments in the skin tend to be irritating. Aggressive topical therapy and some pruritus‐relieving medications will definitely help, but complete resolution requires the elimination of all mite‐related remnants from the skin. With absolute adherence to veterinary recommendations regarding treatment of the pet and in‐contact animals, the itching should start to substantially abate after two weeks, with complete resolution to be anticipated within 6–8 weeks if the pet is not reinfested from its environment.

Setting realistic expectations with clients is critical because ultimate success and client satisfaction will be measured by the owner's perception. Similarly, if you perform a flawless surgery but discharge the animal soiled with faecal matter, the client's perception of the procedure's success may differ significantly from your own. As it is the client who is paying for the service and the one (by definition) who ultimately determines customer satisfaction, the client's interpretation takes precedence. Accordingly, take the time to understand those expectations so you can plan to meet and even exceed them, or be prepared to discuss more realistic expectations with the owner before starting treatment.

## Not viewing the situation in terms of quality of life

Veterinarians might sometimes view dermatologic problems as minor medical issues because often the pet's life is not at risk, but pet owners may not share that same perspective. Pet owners may not know what a diseased liver looks like, but they are certainly aware when their pet keeps them awake at night while scratching, has bald patches in its fur, or harbours offensive odours, rashes, parasites, or skin colour changes associated with a variety of skin diseases. There might also be a certain amount of guilt associated with friends and relatives asking why they have not done a better job of treating their pet's skin ailments, even when the pet owner has done everything they have been instructed to do. Veterinarians and hospital staff would do well to consider what pet owners find stressful, in addition to what might be causing the pet discomfort.

Pet owners are often concerned with the responsibility of successfully managing their pet's problems at home. In many instances, it is stressful for clients to have to regularly administer medications, bathe their pets, apply parasite‐control products on schedule, and other tasks often assigned by hospital staff. Even something as basic as applying ear drops can be a trying experience if the pet demonstrates discomfort, cries, or retreats from the owner, adversely affecting the human–animal bond. People own pets for the positive connection that develops between them and anything that interferes with that is likely to be stressful for both. Veterinary staff should do everything in their power to set clients up for success at home. This includes honestly assessing the recommendations being made and the likelihood that they can be successfully accomplished at home.

When medically prudent to do so, the use of injectable medications is often preferred because it frees the owner up from having to administer medication to their pet. Rather than asking the pet owner to do something at home that might cause their pet discomfort, such as applying ear drops to a painful inflamed ear, the pet might be admitted into the hospital, sedated and appropriately treated and administered anti‐inflammatory therapy or pain management so that the owner is not required to contend with an objectionable experience at home. This might not be possible in all instances, but hospital staff should always strive to make the pet owner's tasks easier at home and to reinforce the human–animal bond at every opportunity by offering such services.

## Not following evidence‐based standards of care

Most pet owners do not want their pets to serve as learning tools, so they appreciate knowing that veterinarians know what they are treating, the chances of success, what problems might be encountered, and the alternatives available should the need arise. Pet owners also appreciate that such knowledge is evidence‐based and reflects established expertise, even if not direct experience. They find comfort in consistent messaging from hospital staff, specialists, and even Internet resources.

Having an evidence‐based action plan assures owners that their pet is not the first to experience this problem, and that there is collective wisdom and experience in how it is best managed (Ackerman 2014).

Standards of care should be created by hospitals to provide this assurance, and to guarantee the best care to all patients and all pet owners, regardless of the individual experience and expertise of the clinician currently treating that pet. Protocols are often used for standardized approaches to basic preventive and acute care (e.g. parasite control, treatment of sarcoptic mange) while care pathways represent a model for approaching problems that might require long‐term or even lifelong care (e.g. atopic dermatitis, demodicosis, pemphigus foliaceus, etc.) (Ackerman *et al*. 2013).

One of the benefits of standards of care is that owners realize that even with a well‐conceived plan, outcomes cannot be guaranteed, and contingency plans may be needed. For example, when treating a dog with bacterial pyoderma, you might select an antibiotic empirically based on the likelihood that the organism is *Staphylococcus pseudintermedius* and that there is a predictive rate of susceptibility and resistance to specific antibiotics. If the pet owner realizes that your plan is based on the likelihood that the organism will be sensitive to the antibiotic selected, but cannot be guaranteed, they can likely also appreciate the need to re‐evaluate the pet at some point and for a bacterial culture and susceptibility test to be performed if there is no evidence of substantial improvement by that time. In fact, following a plan of action allows the owner to feel more engaged in the decision‐making process. They might even participate more fully in the discussion, asking if it might be worthwhile to consider the culture even before starting empirical therapy. This is much more desirable than dispensing treatment, and if it does not seem to be working the owner just decides to get the opinion of another primary‐care veterinarian. Sharing your action plan and thought processes with pet owners can make all the difference in client engagement, consent, and satisfaction.

As the majority of dermatologic cases have underlying disorders, it makes sense to perform tests to try to identify them, so they may be addressed, if possible. Utilizing the basic diagnostic tests, such as skin scrapings, cytology, and fungal cultures, is good medicine and good business. And, if you do have something such as sarcoptic mange on your list of differential diagnoses, it is reasonable to do a parasite‐control trial – even if you do not find parasites on skin scrapings. A properly conducted dietary trial also yields a lot of useful information in the workup of a pruritic animal. However, it is important to have a clear diagnostic plan and to proceed logically. For example, the results from biopsies are more likely to be useful if a thorough history, clinical description and differential diagnoses are provided to the pathologist (images are a nice touch as well). Also, it is worth deciding how and if the test results will potentially change your treatment plan. If they will not alter your decisions, what is the purpose of running the tests?

Because most dermatologic presentations have underlying disorders, you should anticipate recurrence if that underlying problem cannot be permanently corrected. Reaching for a stronger medication just temporarily masks the underlying problem, and clients are quick to recognize this. The answer is to start looking more intently for the primary causes. So, tell clients that you will consider attempting symptomatic therapy at first with an appropriate product, but also tell them that if the problem recurs when the medication is discontinued, more investigation may be warranted before stronger medications are considered. In fact, it is possible that methicillin‐resistant staphylococci might potentially arise in some cases from the unnecessary escalation of antibiotic therapy, among many other theoretical causes (Weese *et al*. 2012).

Dermatologists will tell you that many clients come to referral appointments with a plastic bag full of products that have been ineffective. Clients are often looking for the magic bullet, the one special drug that the dermatologist has access to, but the primary‐care veterinarian does not. The reality is that dermatologists do not have any magic bullets, and rarely rely on one product to address all the needs of their patients. Dermatology patients often have multiple issues to contend with, and treating only one may not be enough to resolve the clinical problem.

For example, many pets with dermatologic issues also have secondary microbial overgrowth (bacterial and/or yeast dermatitis) that needs to be addressed. If those secondary infections are not properly managed, it may be difficult – or impossible – to assess the response to other treatments.

We must also be aware that even well‐accepted theories of cause and effect can and do change over time, and our standards of care must be prepared to change with them. For example, we once thought of atopic dermatitis as a classic Type I hypersensitivity reaction primarily driven by mast‐cell dynamics in response to inhaled allergens. We now know that the major allergen presentation occurs across the skin surface via Langerhans cells and that inflammatory mediators such as interleukin‐31 (IL‐31) and other pruritogenic cytokines are most responsible for the scratching that we see in our allergic patients (Marsella *et al*. 2012). This helps explain why antihistamines are often of limited benefit, and points the way to more targeted therapies that can and should be considered.

When creating standards of care for your hospital, remember to build into the model specific points at which referral to a specialist should take place. The best referral happens as part of a well‐conceived plan, not as an afterthought. It helps to prepare clients early on that the first course of treatment may not resolve the problem, and if so, that you may try other options or recommend the assistance of a specialist. Clients will appreciate your efforts to inform them now, rather than when they are frustrated, upset, and depleted of funds.

For example, dogs with recurrent pyoderma may be controlled adequately with a combination of medications and topical therapies, but including a dermatologist in the decision‐making process early in the course of the condition has many benefits, including helping to determine the underlying causes, and exploring long‐term solutions before chronic changes have occurred in the skin, which might limit the benefits of some treatment options. This can also decrease reliance on medications over the long term, and perhaps stave off the development of antibiotic resistance, so has the potential of saving the owner money, health risks, and effort over time. It is much better for all concerned to have specialists involved early in the course of the disease, when they can play an invaluable role as part of the pet's health care team.

In addition to Standards of Care, Consistency of Care is important in multi‐doctor hospitals. Consistent messaging to clients only occurs when the process of creating standards helps clinicians achieve consensus in their approaches to common clinical entities, so there can be alignment of hospital team members around an established evidence‐based approach, whenever possible.

## Underestimating the role of compliance

When it comes to managing many skin conditions, client compliance is an issue that must be considered.

Before dispensing any medications to owners, be realistic in your expectations for what can be reasonably accomplished at home. Better yet, have that exact conversation with owners, including asking open‐ended questions of their previous experiences, so you can plan your treatment accordingly. Owners are more likely to be compliant treating clinical signs they can judge (e.g. control of scratching or pain), and can be less compliant with things that are not as obvious, such as administering antibiotics orally at specific intervals or remembering to give parasite‐control products on schedule when no parasites are seen.

Because compliance is such an important issue in the resolution of most dermatologic problems, clinicians must recognize this and create treatment plans accordingly. If it is medically prudent to do so, injectable medications should be first‐line therapy, if available, because they offer convenience to the owner and guaranteed compliance. The next preferred medications are ones that can be administered once daily, followed by those that can be administered every 12 h. It is unlikely that oral medications needing to be administered more often than twice daily will be given on schedule other than by the most dedicated of pet owners (Adams, *et al*, 2005). It is also important to remember that administering a medication twice daily is not the same as administering it every 12 h, so even clients that remember to give a medication twice in any given day might not give it at the appropriate interval consistent with the drug's pharmacokinetics. If so, the pet is not receiving the true intended benefit of that medication.

Finally, cost might be one consideration in medication selection, but if the goal is for the pet to actually receive all the benefit of any selected medication, compliance should be the main selection attribute. The most costly medication (in terms of time and medical outcomes) is actually the medication that fails to deliver the anticipated benefits because it was not administered appropriately.

## Lacking realistic product and pricing models

Veterinary hospitals benefit from carrying appropriate products in inventory, pricing them competitively, and tracking compliance to ensure that pets and owners are actually receiving the full benefit of products dispensed and administered.

While clients might want to browse retail store shelves for the best bargains, when it comes to treating their pets with medical issues, they typically want a firm recommendation made by the veterinarian, not a list of possible treatments that the owner can consider. Be prepared to make clear product recommendations, and be prepared to explain why this is the best option for a particular pet. Some clients will always want the least costly option, but veterinarians owe it to their clients to make firm recommendations, based on evidence‐based criteria of what is in the pet's best interest.

Dermatology patients tend to be managed long‐term, so consider which products and pricing models work best for you and your clients to keep that revenue in your practice. It pays to stock products that are priced appropriately for the value delivered, and that have been researched, safety tested, and labelled for use in the species to which they are being administered. However, inventory should be kept lean and there is little need to keep multiple products on the shelf that do essentially the same thing.

## Considering dermatology clients a nuisance

You are in business to serve the needs of your clients. Owing to the nature of dermatologic diseases, you will see most of your dermatologic patients and their owners many times during the year, for many years to come. That gives you a great opportunity to bond with these pet owners and help shape a positive and mutually beneficial veterinarian–client–patient relationship. These are exactly the clients that you should crave for your practice. The next time the pollen count rises and your telephone starts ringing frequently from clients with itchy pets – do not curse – give thanks! If properly counselled, these are probably the most dedicated clients you will have in your practice.
